# l-Iditol

**DOI:** 10.1107/S2414314626008011

**Published:** 2026-08-07

**Authors:** Tomoko Takashita, Yoji Horii, Akari Otabara, Tomohiko Ishii

**Affiliations:** a Graduate School of Science for Creative Emergence Kagawa University, 2217-20 Hayashi-cho Takamatsu Kagawa 761-0396 Japan; Katholieke Universiteit Leuven, Belgium

**Keywords:** crystal structure, hydrogen bonding, rare sugar, sugar alcohol, l-iditol

## Abstract

l-Iditol, C_6_H_14_O_6_, the sugar alcohol corresponding to l-idose, was crystallized from aqueous solution. Colourless block-shaped single crystals suitable for single-crystal X-ray diffraction analysis were obtained. The title compound crystallizes in the monoclinic space group *P*2_1_, with one mol­ecule in the asymmetric unit. In the crystal, all six hy­droxy groups act as donors in O—H⋯O hydrogen bonds, forming a three-dimensional hydrogen-bonded network. The crystal structure of its enanti­omer, d-iditol, has been reported previously [Aza­rnia *et al.* (1972 ▸). *Acta Cryst.* B**28**, 1007–1013].

## Abstract 2

The title compound, l-iditol, was crystallized from aqueous solution. It crystallizes in the monoclinic space group *P*2_1_ with one mol­ecule in the asymmetric unit. The mol­ecules are linked by O—H⋯O hydrogen bonds into a three-dimensional network.

## Graphical abstract

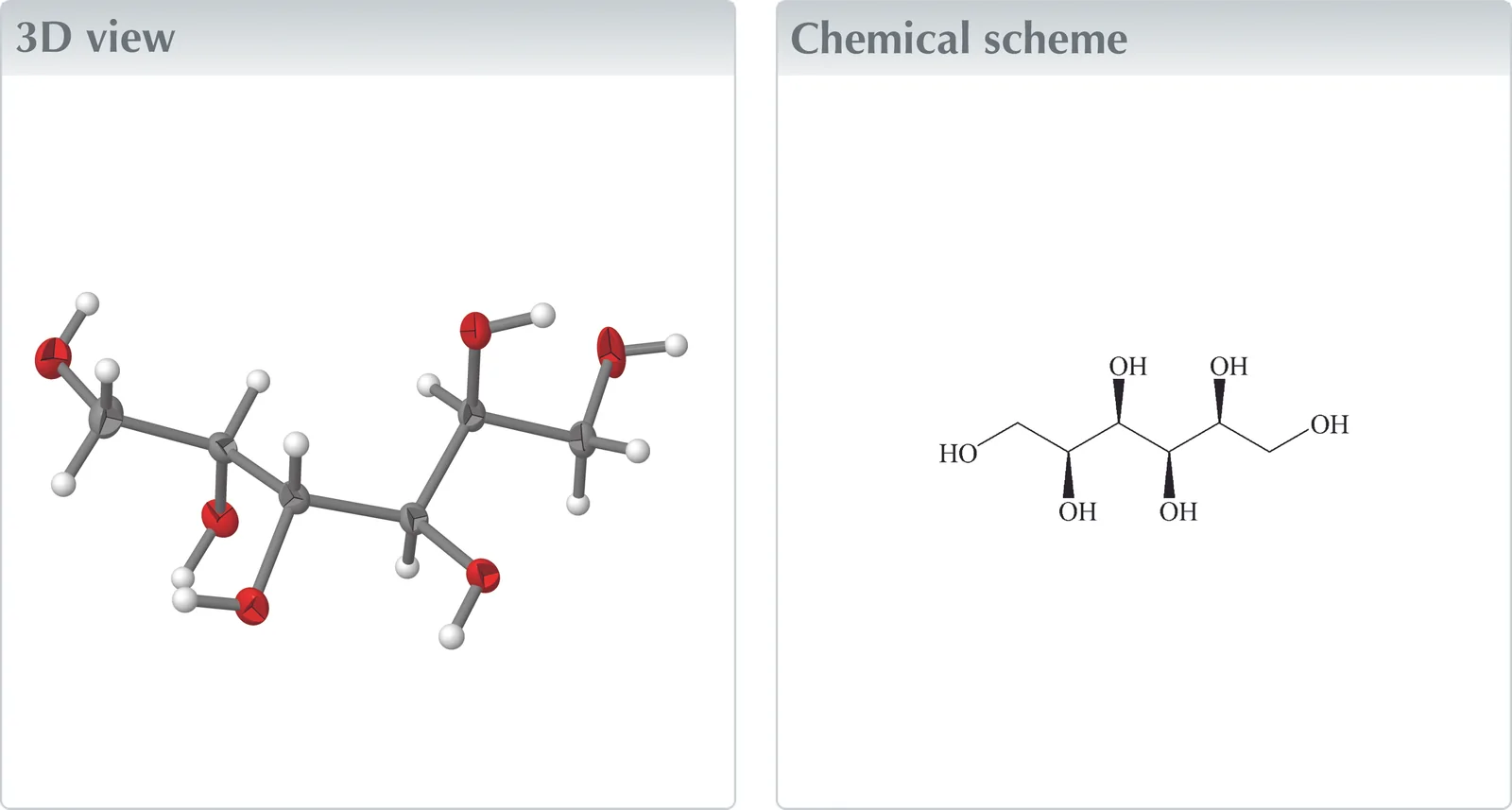

## Structure description

l-Iditol is the sugar alcohol corresponding to the rare sugar l-idose. Systematic bioproduction strategies have expanded the availability of rare hexoses and their corresponding sugar alcohols (Izumori, 2002 ▸). Sugar alcohols possess multiple hy­droxy groups and can therefore form extensive inter­molecular hydrogen-bonding networks. Elucidation of their mol­ecular conformations and crystal packing is important for understanding their solid-state properties.

**Figure 1:**
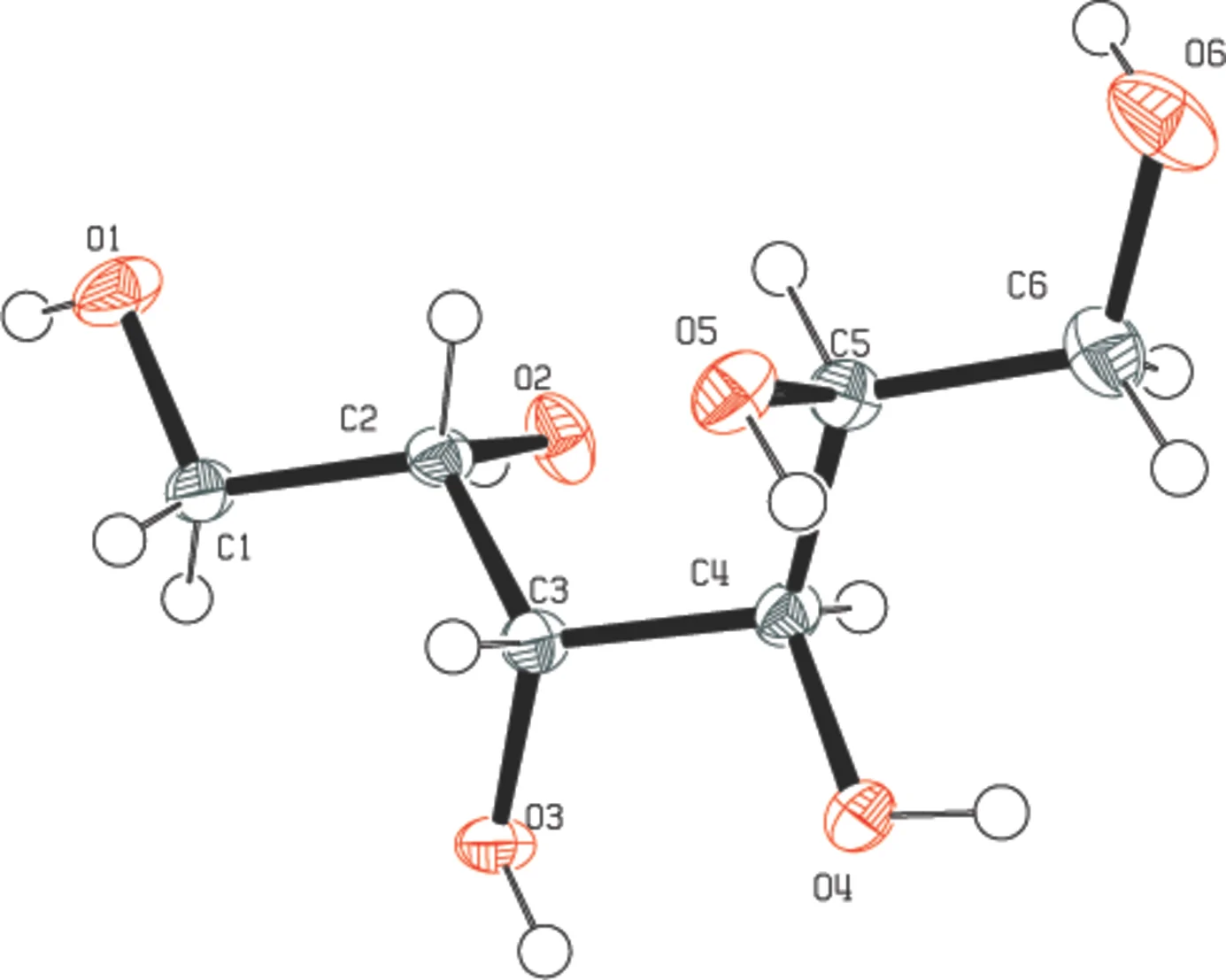
The mol­ecular structure of l-iditol showing the atom-labeling scheme. Displacement ellipsoids are drawn at the 50% probability level, and hydrogen atoms are shown as spheres of arbitrary radii.

The crystal structure of the enanti­omer d-iditol was reported previously by Aza­rnia *et al.* (1972 ▸; CSD refcode IDITOL), and that of racemic d,l-iditol was reported by Kopf *et al.* (1992 ▸; CSD refcode VOMXEA). The present study provides a modern high-precision single-crystal X-ray determination of the corresponding l enanti­omer. The refinement converged at *R*_1_ = 0.0337, and the standard uncertainties of the C—C and C—O bond lengths are in the range 0.003–0.004 Å. Together with the deposited structure-factor data and detailed hydrogen-bond geometry, the present results provide an updated crystallographic description of iditol and specifically document the crystal structure of the l enanti­omer.

l-iditol adopts a twisted, acyclic six-carbon chain conformation (Fig. 1 ▸). The C1—C2—C3—C4, C2—C3—C4—C5 and C3—C4—C5—C6 torsion angles are −179.9 (2), 62.2 (3) and −175.3 (2)°, respectively. Single-crystal X-ray diffraction analysis revealed that it crystallizes in the monoclinic space group *P*2_1_. The asymmetric unit contains one mol­ecule of l-iditol. The stereogenic carbon atoms C2, C3, C4 and C5 have *S*, *R*, *R* and *S* configurations, respectively.

**Figure 2:**
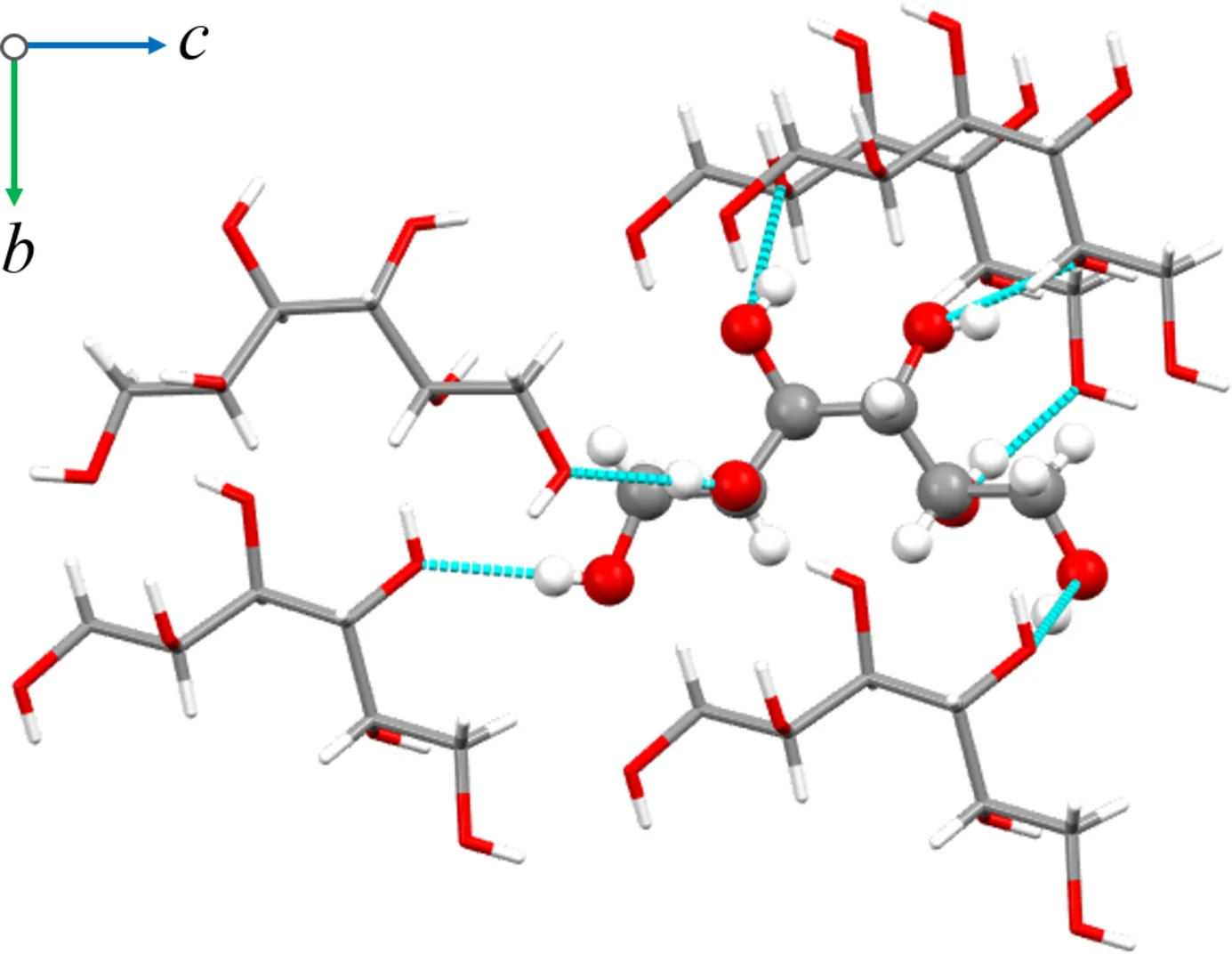
Crystal packing of l-iditol viewed along the *a* axis, showing the three-dimensional hydrogen-bonded network formed by O—H⋯O hydrogen bonds. Hydrogen bonds are shown as dashed lines, and the crystallographic *b*- and *c*-axis directions are indicated.

In the crystal, all six hy­droxy groups act as hydrogen-bond donors, forming six inter­molecular O—H⋯O hydrogen bonds (Table 1 ▸). The donor⋯acceptor distances range from 2.736 (3) to 2.848 (3) Å. These inter­actions connect the mol­ecules into a three-dimensional hydrogen-bonded network. The crystal packing viewed along the *a* axis is shown in Fig. 2 ▸. The inter­molecular hydrogen bonding contributes to the consolidation of the crystal packing.

## Synthesis and crystallization

Commercially available l-iditol (Sigma–Aldrich) was used as received without further purification. The sample was dissolved in water, and the solution was allowed to evaporate slowly at room temperature. Colorless block-shaped single crystals suitable for single-crystal X-ray diffraction analysis were obtained. The absolute configuration was assigned on the basis of the known configuration of the commercially available l-iditol sample.

## Refinement

Crystal data, data collection and structure refinement details are summarized in Table 2 ▸. Although the refined value of the Flack parameter is slightly negative, it is within approximately 1.5 standard uncertainties of zero and is consistent with the absolute configuration assigned from the known configuration of the commercially available l-iditol sample.

## Supplementary Material

Crystal structure: contains datablock(s) I. DOI: 10.1107/S2414314626008011/vm4079sup1.cif

Structure factors: contains datablock(s) I. DOI: 10.1107/S2414314626008011/vm4079Isup2.hkl

Point-by-point response to the Co-editor's comments on manuscript vm4079 (L-iditol). DOI: 10.1107/S2414314626008011/vm4079sup3.docx

Supporting information file. DOI: 10.1107/S2414314626008011/vm4079Isup4.cml

CCDC reference: 2577698

Additional supporting information:  crystallographic information; 3D view; checkCIF report

## Figures and Tables

**Table 1:** Hydrogen-bond geometry (Å, °)

*D*—H⋯*A*	*D*—H	H⋯*A*	*D*⋯*A*	*D*—H⋯*A*
O1—H1⋯O3^i^	0.82	2.04	2.834 (3)	163
O2—H2⋯O6^ii^	0.82	1.95	2.764 (3)	171
O3—H3⋯O5^iii^	0.82	2.01	2.736 (3)	147
O4—H4⋯O2^iv^	0.82	1.98	2.755 (2)	158
O5—H5⋯O1^iii^	0.90 (4)	1.88 (4)	2.766 (3)	170 (3)
O6—H6⋯O3^v^	0.82	2.25	2.848 (3)	130

Symmetry codes: (i) 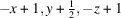; (ii) 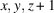; (iii) 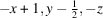; (iv) 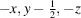; (v) 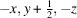.

**Table 2:** Experimental details

Crystal data	
Chemical formula	C_6_H_14_O_6_
*M*_r_	182.17
Crystal system, space group	Monoclinic, *P*2_1_
Temperature (K)	296
*a*, *b*, *c* (Å)	5.8678 (2), 8.3869 (3), 8.1213 (3)
β (°)	93.182 (2)
*V* (Å^3^)	399.05 (2)
*Z*	2
Radiation type	Cu *K*α
μ (mm^−1^)	1.19
Crystal size (mm)	0.10 × 0.10 × 0.10

Data collection	
Diffractometer	Rigaku R-AXIS RAPID
Absorption correction	Multi-scan (*ABSCOR*; Rigaku, 1995 ▸)
*T*_min_, *T*_max_	0.822, 1.000
No. of measured, independent and observed [*I* > 2σ(*I*)] reflections	4219, 1377, 1329
*R*_int_	0.057
(sin θ/λ)_max_ (Å^−1^)	0.602

Refinement	
*R*[*F*^2^ > 2σ(*F*^2^)], *wR*(*F*^2^), *S*	0.034, 0.083, 1.12
No. of reflections	1377
No. of parameters	114
No. of restraints	1
H-atom treatment	H atoms treated by a mixture of independent and constrained refinement
Δρ_max_, Δρ_min_ (e Å^−3^)	0.20, −0.20
Absolute structure	Flack *x* determined using 538 quotients [(*I*^+^)−(*I*^−^)]/[(*I*^+^)+(*I*^−^)] (Parsons *et al.*, 2013 ▸)
Absolute structure parameter	−0.24 (16)

Computer programs: *RAPID-AUTO* (Rigaku, 2009 ▸), *SHELXT2018/2* (Sheldrick, 2015*a* ▸), *SHELXL2018/3* (Sheldrick, 2015*b* ▸) and *OLEX2* (Dolomanov *et al.*, 2009 ▸).

## full crystallographic data

### (2S,3R,4R,5S)-Hexane-1,2,3,4,5,6-hexol Crystal data

**Table:**

C_6_H_14_O_6_	*F*(000) = 196
*M**_r_* = 182.17	*D*_x_ = 1.516 Mg m^−^^3^
Monoclinic, *P*2_1_	Cu *K*α radiation, λ = 1.54187 Å
*a* = 5.8678 (2) Å	Cell parameters from 3800 reflections
*b* = 8.3869 (3) Å	θ = 5.3–68.3°
*c* = 8.1213 (3) Å	µ = 1.19 mm^−^^1^
β = 93.182 (2)°	*T* = 296 K
*V* = 399.05 (2) Å^3^	Block, clear light colourless
*Z* = 2	0.1 × 0.1 × 0.1 mm

### (2S,3R,4R,5S)-Hexane-1,2,3,4,5,6-hexol Data collection

**Table:**

Rigaku R-AXIS RAPID diffractometer	1329 reflections with *I* > 2σ(*I*)
Detector resolution: 10.000 pixels mm^-1^	*R*_int_ = 0.057
ω scans	θ_max_ = 68.2°, θ_min_ = 5.5°
Absorption correction: multi-scan (ABSCOR; Rigaku, 1995)	*h* = −6→7
*T*_min_ = 0.822, *T*_max_ = 1.000	*k* = −10→10
4219 measured reflections	*l* = −9→9
1377 independent reflections	

### (2S,3R,4R,5S)-Hexane-1,2,3,4,5,6-hexol Refinement

**Table:**

Refinement on *F*^2^	H atoms treated by a mixture of independent and constrained refinement
Least-squares matrix: full	*w* = 1/[σ^2^(*F*_o_^2^) + (0.032*P*)^2^ + 0.0743*P*] where *P* = (*F*_o_^2^ + 2*F*_c_^2^)/3
*R*[*F*^2^ > 2σ(*F*^2^)] = 0.034	(Δ/σ)_max_ < 0.001
*wR*(*F*^2^) = 0.083	Δρ_max_ = 0.20 e Å^−^^3^
*S* = 1.12	Δρ_min_ = −0.20 e Å^−^^3^
1377 reflections	Extinction correction: SHELXL 2018/3 (Sheldrick, 2015b), Fc^*^=kFc[1+0.001xFc^2^λ^3^/sin(2θ)]^-1/4^
114 parameters	Extinction coefficient: 0.165 (10)
1 restraint	Absolute structure: Flack *x* determined using 538 quotients [(*I*^+^)-(*I*^-^)]/[(*I*^+^)+(*I*^-^)] (Parsons *et al.*, 2013)
Primary atom site location: dual	Absolute structure parameter: −0.24 (16)
Hydrogen site location: mixed	

### (2S,3R,4R,5S)-Hexane-1,2,3,4,5,6-hexol Special details

**Table:**

Geometry. All esds (except the esd in the dihedral angle between two l.s. planes) are estimated using the full covariance matrix. The cell esds are taken into account individually in the estimation of esds in distances, angles and torsion angles; correlations between esds in cell parameters are only used when they are defined by crystal symmetry. An approximate (isotropic) treatment of cell esds is used for estimating esds involving l.s. planes.
Refinement. The structure was solved using SHELXT (Sheldrick, 2015*a*) and refined on F^2^ by full-matrix least-squares methods using *SHELXL* (Sheldrick, 2015*b*). All non-hydrogen atoms were refined anisotropically. The hydroxy hydrogen atom H5 was located in a difference-Fourier map and refined freely. All other hydrogen atoms were placed in geometrically calculated positions and refined using constrained models. The final refinement gave *R*_1_ = 0.0337 for reflections with *I* > 2σ(*I*) and w*R*_2_ = 0.0829 for all data. The maximum and minimum residual electron densities were 0.20 and -0.20 e Å^-3^, respectively. The Flack parameter was -0.24 (16), determined using 538 quotients of the type [(I^+^) - (I^-^)]/[(I^+^) + (I^-^)] (Parsons *et al.*, 2013). Although the refined value is slightly negative, it is within approximately 1.5 standard uncertainties of zero and is consistent with the absolute configuration assigned from the known configuration of the commercially available *L*-iditol sample.

### (2S,3R,4R,5S)-Hexane-1,2,3,4,5,6-hexol Fractional atomic coordinates and isotropic or equivalent isotropic displacement parameters (Å^2^)

**Table:**

	*x*	*y*	*z*	*U*_iso_*/*U*_eq_	
O1	0.4782 (4)	0.8448 (2)	0.4118 (2)	0.0308 (6)	
H1	0.503187	0.855873	0.511527	0.037*	
O2	0.0813 (3)	0.6622 (2)	0.2718 (2)	0.0251 (5)	
H2	0.078439	0.670204	0.372303	0.030*	
O3	0.3411 (3)	0.3703 (2)	0.2584 (2)	0.0198 (5)	
H3	0.365277	0.284701	0.213852	0.024*	
O4	0.2410 (3)	0.3107 (2)	−0.0653 (2)	0.0230 (5)	
H4	0.152240	0.286824	−0.142921	0.028*	
O5	0.4315 (3)	0.5928 (2)	−0.2005 (2)	0.0216 (5)	
H5	0.466 (6)	0.505 (5)	−0.258 (4)	0.044 (11)*	
O6	0.0239 (4)	0.7005 (3)	−0.3950 (2)	0.0334 (6)	
H6	−0.004973	0.782453	−0.345233	0.040*	
C1	0.4802 (5)	0.6794 (3)	0.3717 (3)	0.0194 (6)	
H1A	0.436997	0.616425	0.465156	0.023*	
H1B	0.631996	0.647182	0.343478	0.023*	
C2	0.3120 (4)	0.6535 (3)	0.2265 (3)	0.0168 (6)	
H2A	0.335094	0.739105	0.146973	0.020*	
C3	0.3538 (4)	0.4961 (3)	0.1405 (3)	0.0158 (6)	
H3A	0.508825	0.497853	0.101329	0.019*	
C4	0.1871 (4)	0.4652 (3)	−0.0076 (3)	0.0159 (6)	
H4A	0.030915	0.464981	0.029349	0.019*	
C5	0.2062 (5)	0.5907 (3)	−0.1429 (3)	0.0176 (6)	
H5A	0.179351	0.694965	−0.093078	0.021*	
C6	0.0294 (5)	0.5697 (4)	−0.2842 (3)	0.0257 (7)	
H6A	0.062713	0.473021	−0.343878	0.031*	
H6B	−0.119857	0.557352	−0.240093	0.031*	

### (2S,3R,4R,5S)-Hexane-1,2,3,4,5,6-hexol Atomic displacement parameters (Å^2^)

**Table:**

	*U*^11^	*U*^22^	*U*^33^	*U*^12^	*U*^13^	*U*^23^
O1	0.0586 (14)	0.0176 (11)	0.0153 (9)	−0.0104 (10)	−0.0058 (9)	−0.0010 (8)
O2	0.0237 (10)	0.0349 (12)	0.0161 (8)	0.0088 (9)	−0.0028 (7)	−0.0053 (8)
O3	0.0318 (10)	0.0126 (9)	0.0150 (9)	0.0036 (7)	0.0005 (7)	0.0004 (7)
O4	0.0313 (11)	0.0153 (9)	0.0214 (9)	0.0014 (8)	−0.0066 (7)	−0.0058 (8)
O5	0.0233 (10)	0.0194 (9)	0.0224 (10)	−0.0052 (8)	0.0047 (8)	−0.0020 (8)
O6	0.0435 (13)	0.0402 (13)	0.0168 (10)	0.0142 (10)	0.0035 (9)	0.0063 (10)
C1	0.0285 (14)	0.0152 (15)	0.0141 (12)	−0.0017 (11)	−0.0026 (11)	0.0000 (10)
C2	0.0239 (13)	0.0149 (13)	0.0114 (11)	−0.0002 (11)	0.0003 (10)	0.0018 (10)
C3	0.0193 (12)	0.0150 (13)	0.0130 (11)	0.0000 (10)	0.0014 (9)	−0.0001 (9)
C4	0.0190 (12)	0.0142 (13)	0.0148 (12)	−0.0012 (10)	0.0015 (10)	−0.0017 (9)
C5	0.0223 (13)	0.0169 (12)	0.0136 (12)	0.0024 (11)	0.0019 (10)	−0.0018 (10)
C6	0.0276 (15)	0.0305 (17)	0.0188 (13)	0.0007 (13)	−0.0015 (11)	0.0027 (12)

### (2S,3R,4R,5S)-Hexane-1,2,3,4,5,6-hexol Geometric parameters (Å, º)

**Table:**

O1—H1	0.8200	C1—H1B	0.9700
O1—C1	1.425 (3)	C1—C2	1.511 (3)
O2—H2	0.8200	C2—H2A	0.9800
O2—C2	1.424 (3)	C2—C3	1.521 (3)
O3—H3	0.8200	C3—H3A	0.9800
O3—C3	1.430 (3)	C3—C4	1.529 (3)
O4—H4	0.8200	C4—H4A	0.9800
O4—C4	1.420 (3)	C4—C5	1.530 (4)
O5—H5	0.90 (4)	C5—H5A	0.9800
O5—C5	1.427 (3)	C5—C6	1.514 (4)
O6—H6	0.8200	C6—H6A	0.9700
O6—C6	1.418 (4)	C6—H6B	0.9700
C1—H1A	0.9700		
			
C1—O1—H1	109.5	C2—C3—H3A	108.2
C2—O2—H2	109.5	C2—C3—C4	113.3 (2)
C3—O3—H3	109.5	C4—C3—H3A	108.2
C4—O4—H4	109.5	O4—C4—C3	105.6 (2)
C5—O5—H5	114 (2)	O4—C4—H4A	109.2
C6—O6—H6	109.5	O4—C4—C5	111.3 (2)
O1—C1—H1A	110.2	C3—C4—H4A	109.2
O1—C1—H1B	110.2	C3—C4—C5	112.3 (2)
O1—C1—C2	107.7 (2)	C5—C4—H4A	109.2
H1A—C1—H1B	108.5	O5—C5—C4	110.5 (2)
C2—C1—H1A	110.2	O5—C5—H5A	107.3
C2—C1—H1B	110.2	O5—C5—C6	111.2 (2)
O2—C2—C1	112.4 (2)	C4—C5—H5A	107.3
O2—C2—H2A	107.5	C6—C5—C4	112.9 (2)
O2—C2—C3	110.1 (2)	C6—C5—H5A	107.3
C1—C2—H2A	107.5	O6—C6—C5	112.4 (2)
C1—C2—C3	111.6 (2)	O6—C6—H6A	109.1
C3—C2—H2A	107.5	O6—C6—H6B	109.1
O3—C3—C2	108.48 (18)	C5—C6—H6A	109.1
O3—C3—H3A	108.2	C5—C6—H6B	109.1
O3—C3—C4	110.3 (2)	H6A—C6—H6B	107.8
			
O1—C1—C2—O2	−73.8 (3)	O5—C5—C6—O6	−64.8 (3)
O1—C1—C2—C3	162.0 (2)	C1—C2—C3—O3	57.2 (3)
O2—C2—C3—O3	−68.3 (2)	C1—C2—C3—C4	−179.9 (2)
O2—C2—C3—C4	54.6 (2)	C2—C3—C4—O4	−176.3 (2)
O3—C3—C4—O4	−54.5 (2)	C2—C3—C4—C5	62.2 (3)
O3—C3—C4—C5	−176.0 (2)	C3—C4—C5—O5	59.4 (3)
O4—C4—C5—O5	−58.8 (3)	C3—C4—C5—C6	−175.3 (2)
O4—C4—C5—C6	66.5 (3)	C4—C5—C6—O6	170.3 (2)

### (2S,3R,4R,5S)-Hexane-1,2,3,4,5,6-hexol Hydrogen-bond geometry (Å, º)

**Table:**

*D*—H···*A*	*D*—H	H···*A*	*D*···*A*	*D*—H···*A*
O1—H1···O3^i^	0.82	2.04	2.834 (3)	163
O2—H2···O6^ii^	0.82	1.95	2.764 (3)	171
O3—H3···O5^iii^	0.82	2.01	2.736 (3)	147
O4—H4···O2^iv^	0.82	1.98	2.755 (2)	158
O5—H5···O1^iii^	0.90 (4)	1.88 (4)	2.766 (3)	170 (3)
O6—H6···O3^v^	0.82	2.25	2.848 (3)	130

Symmetry codes: (i) −*x*+1, *y*+1/2, −*z*+1; (ii) *x*, *y*, *z*+1; (iii) −*x*+1, *y*−1/2, −*z*; (iv) −*x*, *y*−1/2, −*z*; (v) −*x*, *y*+1/2, −*z*.
