# Supplementary material for: l-Iditol
Source: IUCrdata. 2026 Aug 7;11(Pt 8):x260801. doi: 10.1107/S2414314626008011 (PMC13548375; doi:10.1107/S2414314626008011)
Supplement: Supplementary file 1 [file x-11-x260801-sup3.docx]

Response to Co-editor Professor L. Van Meervelt

Manuscript ID: vm4079

Title: L-iditol

We thank Professor L. Van Meervelt for the careful assessment of our manuscript and for the helpful comments.

**Comment 1:**

The authors need to provide a better justification why they are publishing an enantiomer of a known structure; presumably the precision is higher than the 1972 study?

**Response:**

The submitted manuscript already cited the previously reported crystal structure of D-iditol by Azarnia et al. (1972), as well as that of racemic D,L-iditol reported by Kopf et al. (1992). To address the Editor's comment, we have expanded the relevant discussion to clarify the significance of the present determination. The revised text now states that the present study provides a modern high-precision single-crystal X-ray determination of the L enantiomer. The refinement converged at R1 = 0.0337, and the standard uncertainties of the C—C and C—O bond lengths are in the range 0.003–0.004 Å. We have also noted that the present crystallographic dataset includes deposited structure factors and detailed hydrogen-bond geometry.

**Comment 2:**

Figure 1: use larger atom labels and move labels to avoid overlap with the atom.

**Response:**

The atom labels in Figure 1 have been enlarged and repositioned to improve their legibility and avoid overlap with the atoms. A revised Figure 1 has been uploaded.

**Comment 3:**

Figure 2: also add the unit cell or unit cell directions

**Response:**

The crystallographic b- and c-axis directions have been added to Figure 2. The figure caption and the corresponding manuscript text have also been revised to state that the crystal packing is viewed along the a axis. A revised Figure 2 has been uploaded.

Yours sincerely,

Tomoko TAKASHITA

Graduate School of Science for Creative Emergence, Kagawa University, Japan
